# Impact of T2DM on right ventricular systolic dysfunction and interventricular interactions in patients with essential hypertension: evaluation using CMR tissue tracking

**DOI:** 10.1186/s12933-022-01678-3

**Published:** 2022-11-09

**Authors:** Xue-Ming Li, Wei-Feng Yan, Li Jiang, Ke Shi, Yan Ren, Pei-Lun Han, Li-Qing Peng, Ying-Kun Guo, Zhi-Gang Yang

**Affiliations:** 1 Department of Radiology, West China Hospital, Sichuan University, 37# Guo Xue Xiang, Chengdu, 610041 Sichuan People’s Republic of China; 2 Laboratory of Cardiovascular Diseases, Regenerative Medicine Research Center, West China Hospital, Sichuan University, 37# Guo Xue Xiang, Chengdu, 610041 Sichuan People’s Republic of China; 3 Department of Endocrinology and Metabolism, West China Hospital, Sichuan University, 37# Guo Xue Xiang, Chengdu, 610041 Sichuan People’s Republic of China; 4 Department of Radiology, Key Laboratory of Birth Defects and Related Diseases of Women and Children of Ministry of Education, West China Second University Hospital, Sichuan University, 20# South Renmin Road, Chengdu, 610041 Sichuan People’s Republic of China

**Keywords:** Hypertension, Type 2 diabetes, Left ventricle, Right ventricle, Magnetic resonance imaging, Strain, Systolic dysfunction, Interventricular coupling

## Abstract

**Background:**

Previous studies reported that there was right ventricular (RV) systolic dysfunction in patients with hypertension. The aim of this study was to evaluate the impact of type 2 diabetes mellitus (T2DM) on RV systolic dysfunction and interventricular interactions using cardiac magnetic resonance feature tracking (CMR-FT) in patients with essential hypertension.

**Methods and methods:**

Eighty-five hypertensive patients without T2DM [HTN(T2DM −)], 58 patients with T2DM [HTN(T2DM +)] and 49 normal controls were included in this study. The biventricular global radial, circumferential and longitudinal peak strains (GRS, GCS, GLS, respectively) and RV regional strains at the basal-, mid- and apical-cavity, were calculated with CMR-FT and compared among controls and different patient groups. Backward stepwise multivariable linear regression analyses were used to determine the effects of T2DM and left ventricular (LV) strains on RV strains.

**Results:**

The biventricular GLS and RV apical longitudinal strain deteriorated significantly from controls, through HTN(T2DM-), to HTN(T2DM +) groups. RV middle longitudinal strain in patient groups were significantly reduced, and LV GRS and GCS and RV basal longitudinal strain were decreased in HTN(T2DM +) but preserved in HTN(T2DM-) group. Multivariable regression analyses adjusted for covariates demonstrated that T2DM was independently associated with LV strains (LV GRS: β = − 4.278, p = 0.004, model R^2^ = 0.285; GCS: β = 1.498, p = 0.006, model R^2^ = 0.363; GLS: β = 1.133, p = 0.007, model R^2^ = 0.372) and RV GLS (β = 1.454, p = 0.003, model R^2^ = 0.142) in hypertension. When T2DM and LV GLS were included in the multiple regression analysis, both T2DM and LV GLS (β = 0.977 and 0.362, p = 0.039 and < 0.001, model R^2^ = 0.224) were independently associated with RV GLS.

**Conclusions:**

T2DM exacerbates RV systolic dysfunction in patients with hypertension, which may be associated with superimposed LV dysfunction by coexisting T2DM and suggests adverse interventricular interactions.

## Introduction

Type 2 diabetes mellitus (T2DM) and essential hypertension commonly coexist, and coexisting T2DM further increases the risk of adverse cardiovascular events in patients with hypertension [1]. Studies on the effects of hypertension and T2DM on the heart primarily focused on the left ventricle and found that these conditions lead to left ventricular (LV) structural and functional abnormalities [2–4]. However, their effects on the right ventricle were not extensively examined, and relatively few studies existed [5–7]. Recent studies showed that right ventricular (RV) dysfunction was an important indicator of cardiac morbidity and mortality in a variety of cardiovascular diseases [8, 9]. Therefore, it is of great clinical importance to evaluate the synergistic effects of T2DM and hypertension on the right ventricle.

Echocardiography is widely used for RV evaluations in clinical settings. However, the location and complex anatomical structure of right ventricle are challenging [10], and the acoustic window in many patients limits imaging due to its angle dependence. Compared with echocardiography, cardiac magnetic resonance (CMR) imaging is considered the gold standard for accurate measurement of RV size and function, especially when the acoustic window is poor [11]. Echocardiography speckle tracking and CMR feature tracking (CMR-FT) can directly evaluate the global and regional myocardial deformation, which help detect subclinical myocardial dysfunction [12].

Some previous studies have demonstrated RV systolic dysfunction in patients with hypertension using echocardiography-based myocardial deformation measurements [13–17]. To the best of our knowledge, there was limited study using myocardial deformation to evaluate the interaction between ventricles [7], and no studies investigated the impact of T2DM on RV dysfunction in patients with hypertension. Therefore, the aim of this study was to evaluate the effects of T2DM on subclinical RV systolic dysfunction in patients with hypertension using CMR-FT and examine the coupling relationship between the right and left ventricles.

## Materials and methods

### Study population

A total of 285 adult patients with essential hypertension who underwent CMR examination in our hospital from January 2016 to December 2021 were enrolled and divided into groups with or without T2DM [HTN(T2DM +) and HTN(T2DM-)]. Hypertension was defined as systolic blood pressure (SBP) ≥ 140 mmHg and/or diastolic blood pressure (DBP) ≥ 90 mmHg at rest measured on more than two occasions or the use of antihypertensive drugs. The diagnosis of T2DM was based on the guidelines of the American Diabetes Association [18]. The exclusion criteria were patients with coronary heart disease (myocardial infarction, percutaneous coronary intervention and/or coronary artery bypass grafting), symptoms of heart failure, left or right ventricular ejection fraction < 50%, moderate to severe valvular disease, congenital heart disease, cardiomyopathy, atrial fibrillation, severe hepatopulmonary dysfunction, severe renal insufficiency (eGFR < 30 mL/1.73 mm^2^), history of chemo- or radiotherapy, inflammatory disease and myocarditis. Patients with poor image quality for left or right ventricle and who were unmatched for age and sex were also excluded. Finally, 143 patients were eligible for this study, including 85 patients with HTN(T2DM-) (46 males, 39 females, mean age 57.0 ± 12.4 years) and 58 patients with HTN(T2DM +) (31 males, 27 females, mean age 59.5 ± 9.2 years). Forty-nine age- and sex-matched healthy individuals (26 males, 23 females, mean age 56.6 ± 10.1 years) with no history of impaired glucose tolerance, electrocardiogram (ECG) abnormalities, symptoms of cardiovascular disease or cardiovascular abnormalities detected using CMR (reduced EF in both ventricles, abnormal ventricular motion, valvular stenosis, or regurgitation) were included as the control group. This study was approved by the Biomedical Research Ethics Committee of our hospital and conducted in accordance with the Declaration of Helsinki.

### Image acquisition

All CMR examinations were performed in the supine position using a 3.0 T whole body magnetic resonance scanner TrioTim or MAGNETOM Skyra (Siemens Medical Solutions, Erlangen, Germany) equipped with 32-channel body phased array coils and standard ECG trigger equipment. Balanced steady-state free precession (b-SSFP) cine images were acquired using a retrospective vector ECG gating technique at the end of inspiratory breath holding, and twenty-five frames were reconstructed per breath-hold acquisition. Standard short-axis, long-axis two- and four-chamber cine images were obtained, which covered the entire left and right ventricles. The following scanning parameters were used: repetition time [TR] 2.81 ms or 3.4 ms, echo time [TE] 1.22 ms, flip angle 40° or 50°, slice thickness 8 mm, field of view [FOV] 250 × 300 mm^2^ or 340 × 285mm^2^, and matrix 208 × 139 or 256 × 166.

### Image analysis

The CMR images were uploaded to offline commercial software (Cvi42, v.5.11.2; Circle Cardiovascular Imaging, Calgary, Canada) and analyzed by two radiologists who were blinded to the clinical data of the subjects. Both radiologists had more than three years of experience in CMR imaging.

**Fig. 1:**
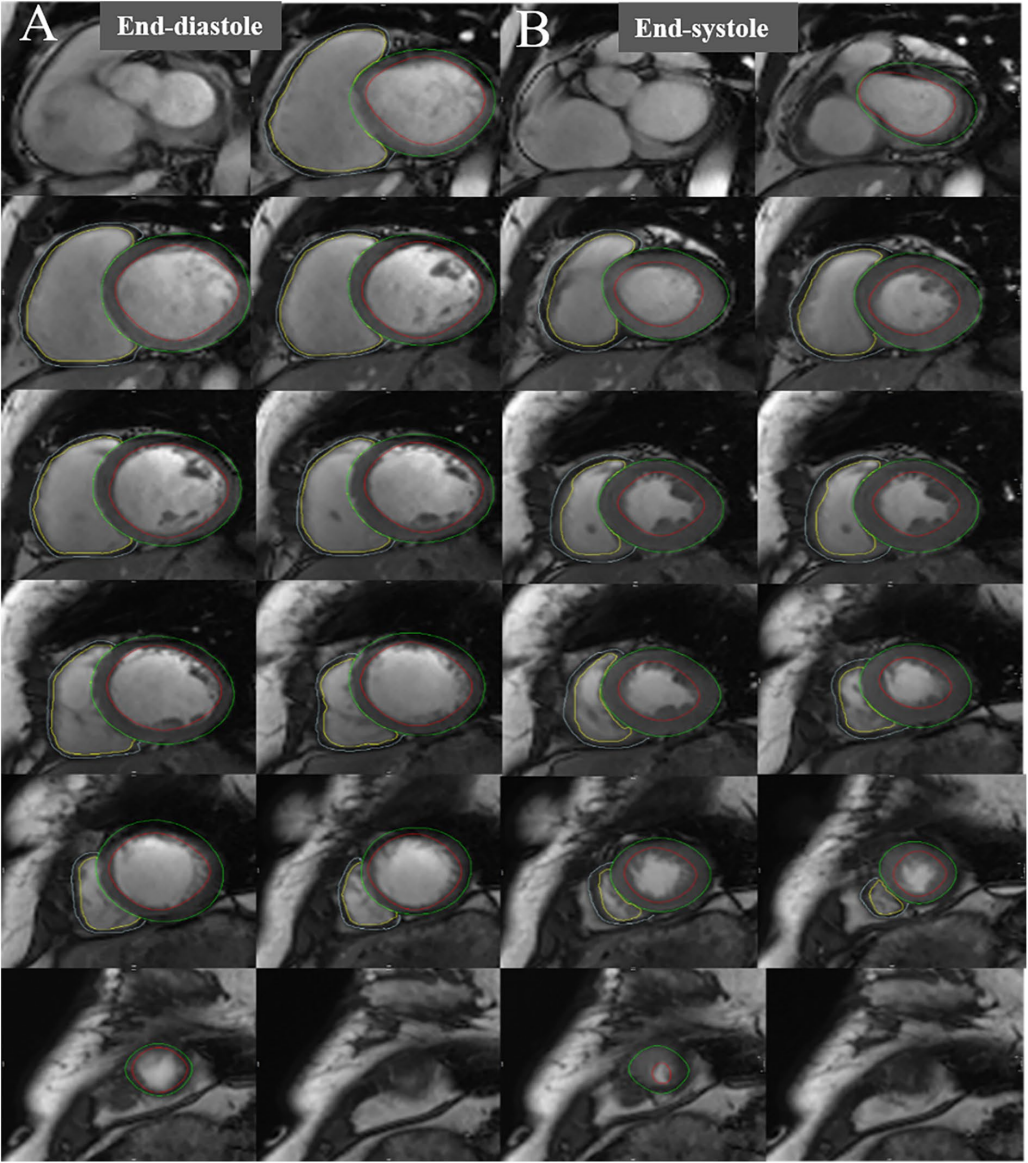
Postprocessing analysis for evaluations of ventricular volumes and masses in both ventricles. The endocardial and epicardial contours of both ventricles were outlined in end-diastole (**A**) and end-systole (**B**) of short axis cine images to calculate ventricular volumes and myocardial masses. The yellow and blue curves represent the endocardial and epicardial contours of the right ventricle, and the red and green curves represent those of the left ventricle, respectively

The endo- and epicardial contours of both ventricles were manually delineated at the end-diastolic and end-systolic phases on the short-axis cine images in the Short-3D module, and the morphological and functional parameters were calculated automatically (Fig. 1), including LV and RV end-diastolic volume (EDV), end-systolic volume (ESV), stroke volume (SV), cardiac output (CO), ejection fraction (EF) and myocardial masses (M). The papillary muscle and trabeculae were excluded from myocardial masses but included in ventricular volume analyses. The body surface area (BSA) was calculated using the Mosteller formula [19], and the volumes and masses of both ventricles were indexed for BSA (EDVI, ESVI, SVI, CI, MI, respectively). LV and RV remodeling indices (LVRI and RVRI, respectively) were calculated as LVM/LVEDV and RVM/RVEDV.

**Fig. 2:**
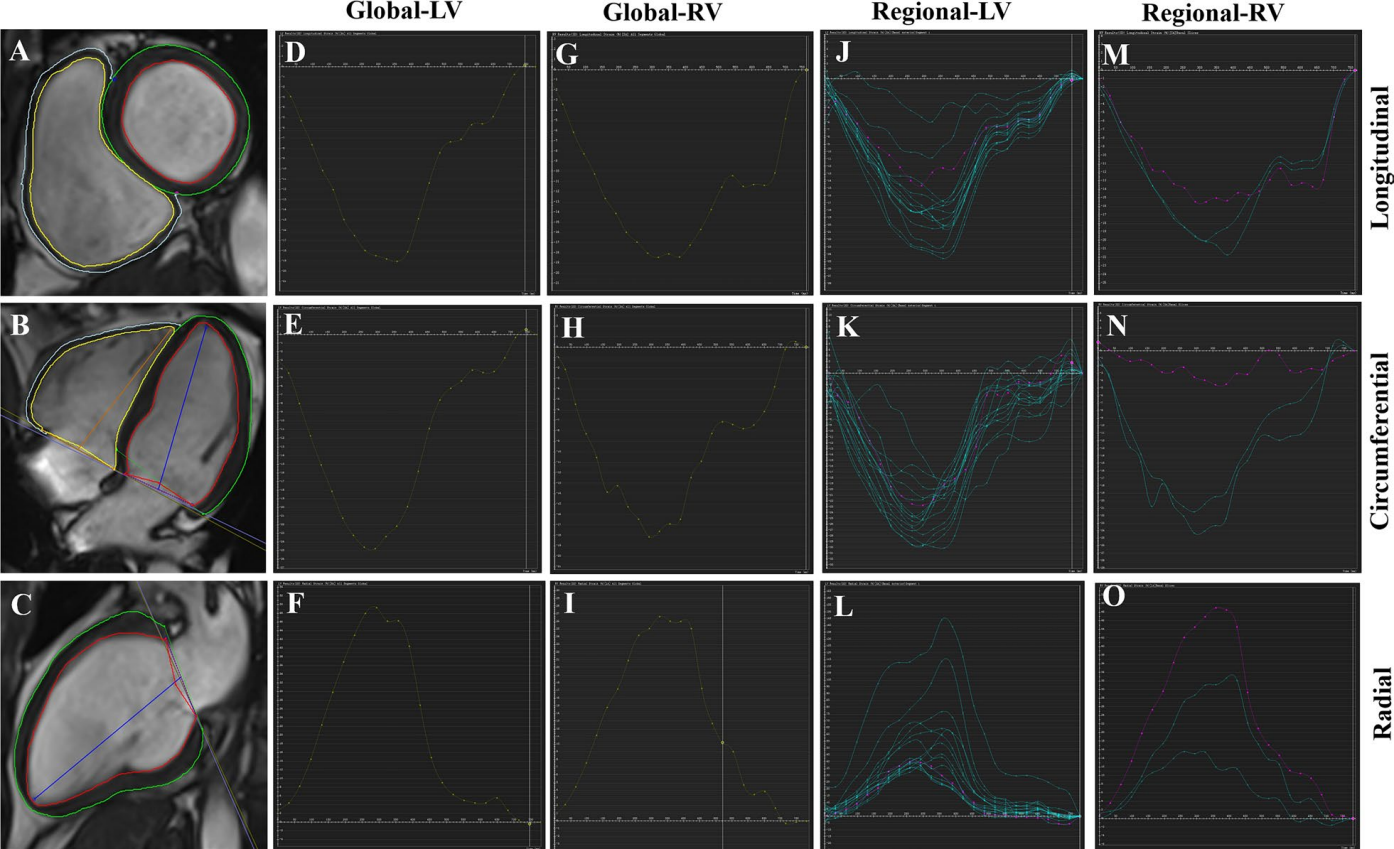
Examples of evaluations of left and right ventricular global and regional strains using CMR-FT. **A**–**C** The endo- and epicardial contours of the left and right ventricles are delineated on standard cardiac short-axis, four-chamber, and two-chamber planes in end diastole. **D**–**I** Global longitudinal, circumferential and radial strains of the left (**D**–**F**) and right (**G**–**I**) ventricles. **J**–**L** Regional longitudinal, circumferential and radial strains of the AHA 16-segment model in the left ventricle. **M**–**O** Regional longitudinal, circumferential and radial strains of the right ventricle in the basal, middle and apical cavities. The yellow and blue curves represent the endo- and epicardial contours of the right ventricle, and the red and green curves represent those of left ventricle

**Fig. 3:**
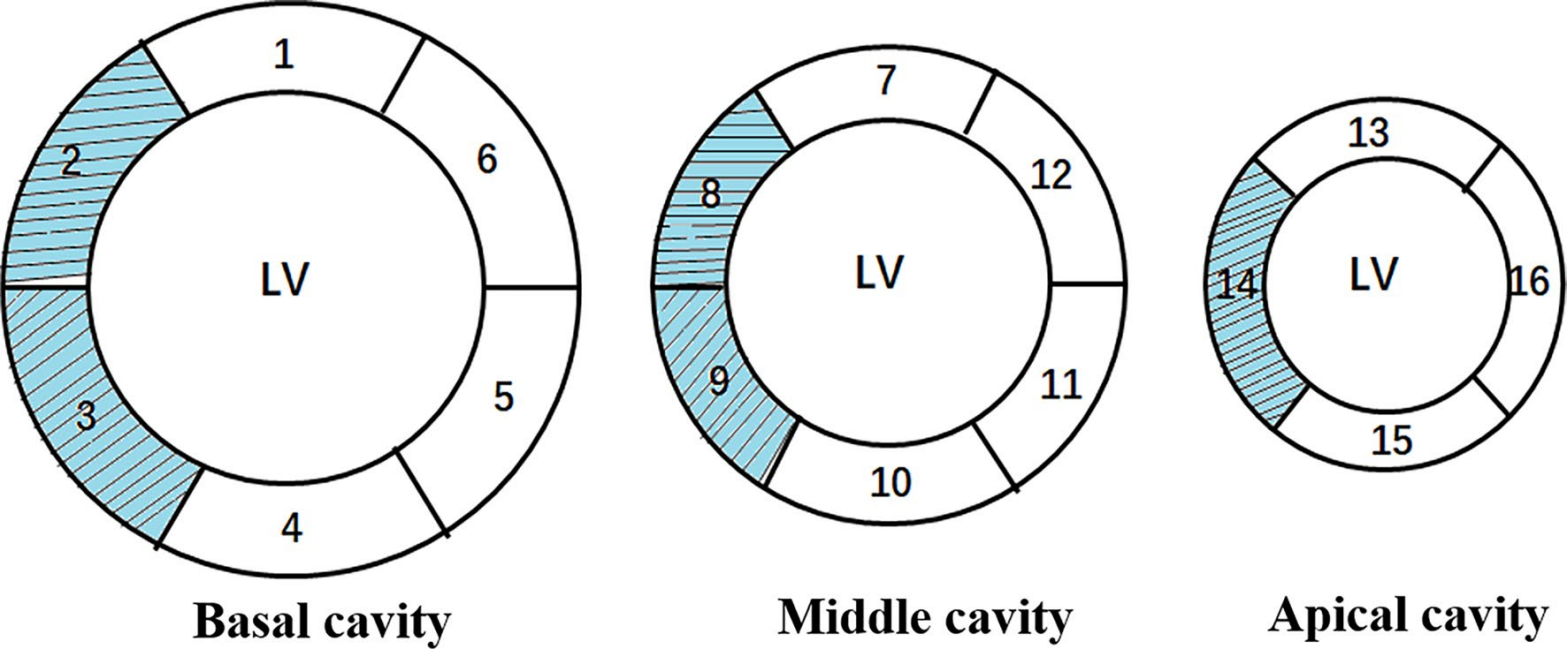
The 16-segment model of left ventricle according to American Heart Association (AHA). Segments 2, 3, 8, 9 and 14 represent the interventricular septum

The short-axis, long-axis four- and two-chamber cine images were loaded into the tissue tracking module to evaluate the myocardial strain of both ventricles. The endocardium and epicardium of both ventricles were manually outlined at end diastole (reference phase) after careful exclusion of papillary muscles and trabeculae. The RV insertion points were marked to allow accurate segmentation according to the standard American Heart Association (AHA) segment [20], and the extent of both ventricles was defined in the long-axis views. The biventricular global radial (GRS), circumferential (GCS) and longitudinal peak strains (GLS), RV regional strains (including the basal, middle, and apical cavities) and LV segmental strains were generated automatically (Fig. 2). According to the 16-segment model of the AHA, segments 2, 3, 8, 9 and 14 represented the area of interventricular septum (IVS) (Fig. 3).

### Reproducibility of RV strains

To evaluate the interobserver variability, 30 cases were randomly selected, and the RV global and regional strains were independently analyzed by two radiologists in a double-blinded manner. The intraobserver variability was analyzed by comparing the measurements of the same subjects by one of the radiologists at an interval of one month.

### Statistical analysis

All statistical analyses were performed using SPSS (version 23.0, IBM, Armonk, New York, USA). Categorical data are expressed as frequencies (percentages) and were compared using the chi-squared or Fisher’s exact test as appropriate. The Shapiro–Wilk test was performed to evaluate the normality of continuous variables. Data with a normal distribution are expressed as means ± standard deviation, and data with non-normal distribution are expressed as medians (25–75% interquartile range). One-way analysis of variance (one-way ANOVA) was used to compare the baseline clinical characteristics and regional myocardial strains of right ventricle and IVS. Comparisons of CMR-derived ventricular volumetrics and global strains were evaluated using analysis of covariance (ANCOVA) after adjusting for age, sex, body mass index (BMI) and heart rate followed by Bonferroni’s post-hoc test. Pearson or Spearman’s correlation coefficient was used to analyze the correlations between CMR-derived RV function and both ventricular volumetrics, LV global strains and regional strains of IVS in patients with hypertension. Multivariable stepwise backward linear regression analyses were performed to determine the predictors for both ventricular global strains in the entire and hypertensive populations and the independent predictive ability of LV strains for RV strains. The intraobserver and interobserver variabilities of RV global and regional deformation were analyzed using the intraclass correlation coefficient (ICC). Two-tailed p < 0.05 was considered statistically significant.

## Results

### Participants’ clinical characteristics

The baseline clinical characteristics of the participants are shown in Table 1. The BMI, SBP and DBP in both patient groups were significantly higher than the control group (all p ≤ 0.001). The fasting blood glucose in the HTN(T2DM +) group was significantly higher than the HTN(T2DM-) group and controls (all p < 0.001).

**Table 1:** Baseline characteristics of the study population

	Controls	HTN(T2DM-)	HTN(T2DM +)	*P* value
	n = 49	n = 85	n = 58	
Demographics				
Female, n (%)	23(46.9)	39(45.9)	27(46.6)	1.000
Age (year)	56.6 ± 10.1	57.0 ± 12.4	59.5 ± 9.2	0.294
BMI (kg/m^2^)	22.85 ± 3.01	24.88 ± 2.85*	24.64 ± 3.06*	0.001
BSA (m^2^)	1.68 ± 0.19	1.74 ± 0.20	1.70 ± 0.15	0.156
Smoking, n (%)	0	28(37.3)	15(31.3)	0.563
Duration of hypertension (year)	0	7.5 ± 8.7	7.7 ± 8.2	0.875
Duration of diabetes (year)	0	0	8.6 ± 5.3	
Laboratory data				
Fasting blood glucose (mmol/L)	5.65 ± 1.61	5.31 ± 0.84	7.94 ± 2.94*§	< 0.001
Plasma triglycerides (mmol/L)	1.51 ± 0.85	1.89 ± 1.68	1.77 ± 1.57	0.471
Total cholesterol (mmol/L)	4.56 ± 0.89	4.43 ± 1.02	4.15 ± 0.82	0.098
HDL (mmol/L)	1.29 ± 0.32	1.31 ± 0.47	1.20 ± 0.34	0.259
LDL (mmol/L)	2.72 ± 0.78	2.49 ± 0.84	2.38 ± 0.73	0.153
eGFR (mL/min/1.73m^2^)	92.17 ± 17.89	89.890 ± 19.57	87.16 ± 20.80	0.487
Hemodynamic variables				
Heart rate(beats/min)	72.7 ± 11.8	73.8 ± 13.0	73.4 ± 11.1	0.877
SBP (mmHg)	119.7 ± 14.6	139.0 ± 20.8*	135.0 ± 17.1*	< 0.001
DBP (mmHg)	73.7 ± 8.7	86.3 ± 13.9*	81.9 ± 9.8*	< 0.001
Antihypertensive medication				
ACEI/ARB, n (%)	0	37(43.5)	27 (46.6)	0.735
Bta-blocker, n (%)	0	30 (35.3)	19 (32.8)	0.858
Calcium channel blocker, n (%)	0	51 (60.7)	30 (51.7)	0.306
Diuretics, n (%)	0	13 (15.3)	10 (17.2)	0.818
Antidiabetic medication				
Oral, n (%)	0	0	44 (75.9)	
Insulin, n (%)	0	0	16 (27.6)	

Values are mean ± standard deviation, numbers in the brackets are percentage

*eGFR* estimated glomerular filtration rate, *HDL* high-density lipoprotein cholesterol, *LDL* low-density lipoprotein cholesterol, *ACEI* angiotensin converting enzyme inhibitor, *ARB* angiotensin II receptor blocker

^*^p < 0.05 vs. controls

^§^P < 0.05 vs. controls and HTN (T2DM-) group

### Characteristics of biventricular volumetrics in patient groups

Comparisons of left and right ventricular volumetric parameters are shown in Table 2. Compared with controls, the biventricular masses and remodeling indices were significantly increased in both patient groups (all p < 0.001) but were not significantly different between each other. There was no significant difference in biventricular EF, end-diastolic and end-systolic volume indices, stroke volume or cardiac output indices among the groups (all p > 0.05).

**Table 2:** Comparison of left and right volumetric parameters among groups

	Controls	HTN(T2DM-)	HTN(T2DM +)	*P* value
LV geometry and function				
LVEF (%)	64.51 ± 6.61	64.66 ± 5.83	62.25 ± 8.30	0.095
LVEDVI (mL/m^2^)	77.38 ± 11.49	78.01 ± 16.68	78.71 ± 16.84	0.913
LVESVI (mL/m^2^)	27.41 ± 6.95	27.88 ± 8.63	30.49 ± 11.42	0.170
LVSVI (mL/m^2^)	50.09 ± 9.37	49.88 ± 10.28	48.53 ± 9.32	0.665
LV cardiac index (L/min/m^2^)	3.61 ± 0.90	3.66 ± 0.80	3.57 ± 0.71	0.859
LVMI (g/m^2^)	41.91 ± 9.34	54.92 ± 12.59*	54.78 ± 13.76*	< 0.001
LV remodeling index (g/mL)	0.55 ± 0.90	0.73 ± 0.17*	0.72 ± 0.17*	< 0.001
RV geometry and function				
RVEF (%)	58.94 ± 6.72	58.44 ± 6.59	58.26 ± 7.47	0.872
RVEDVI (mL/m^2^)	69.70 ± 12.91	69.25 ± 15.69	69.69 ± 14.27	0.979
RVESVI (mL/m^2^)	28.54 ± 6.93	29.20 ± 9.00	30.03 ± 9.90	0.702
RVSVI (mL/m^2^)	40.16 ± 7.96	40.11 ± 8.90	40.08 ± 7.46	0.999
RV cardiac index (L/min/m^2^)	2.91 ± 0.68	2.98 ± 0.81	2.89 ± 0.73	0.747
RVMI (g/m^2^)	15.06 ± 2.39	17.34 ± 3.06*	17.25 ± 2.81*	< 0.001
RV remodeling index(g/mL)	0.22 ± 0.03	0.25 ± 0.03*	0.24 ± 0.04*	< 0.001

*LV* left ventricular, *RV* right ventricular, *EF* ejection fraction, *EDV* end diastolic volume, *ESV* end systolic volume, *SV* stroke volume, *M* mass, *I* indexed to BSA

^*^p < 0.024 vs. Controls

### Characteristics of global biventricular and regional RV and IVS strains in patient groups

The biventricular GLS and RV apical longitudinal strains were decreased gradually from the controls through HTN(T2DM-) group to the HTN(T2DM +) group (all p < 0.001). The LV GRS (p < 0.001 and = 0.023) and GCS (p = 0.005 and 0.012), and RV basal longitudinal strain (p = 0.013 and 0.003) in patients with HTN(T2DM +) were lower than the HTN(T2DM-) group and controls, but they were not reduced in the HTN(T2DM-) group (all p > 0.05). Compared to controls, longitudinal strain in the middle cavity of the RV was reduced in both patient groups (all p < 0.05). (Table 3).

**Table 3:** Comparison of global strain of both ventricles and regional strain of right ventricle

	Controls	HTN(T2DM-)	HTN(T2DM +)	*P* value
Global myocardial peak strain of LV				
GRS (%)	37.60 ± 8.21	34.65 ± 9.61	30.59 ± 8.64*§	< 0.001
GCS (%)	− 21.12 ± 2.50	− 20.59 ± 3.54	− 18.81 ± 3.35*§	< 0.002
GLS (%)	− 14.74 ± 2.09	− 13.09 ± 2.75*	− 11.68 ± 2.74*§	< 0.001
Global and regional myocardial peak strain of RV				
Radial peak strain (%)				
GRS	32.22 ± 9.57	36.03 ± 15.13	31.89 ± 10.04	0.083
Basal cavity	45.63 ± 16.80	48.43 ± 23.16	40.57 ± 17.46	0.076
Mid cavity	33.99 ± 15.11	38.04 ± 19.42	36.05 ± 17.81	0.448
Apical cavity	26.90 ± 12.64	30.41 ± 22.96	30.46 ± 20.49	0.569
Circumferential peak strain (%)				
GCS	− 13.94 ± 3.49	− 12.27 ± 4.03	− 12.23 ± 3.88	0.052
Basal cavity	− 3.50 ± 7.29	− 2.56 ± 7.76	− 2.46 ± 7.12	0.730
Mid cavity	− 17.10 ± 3.84	− 15.13 ± 4.64	− 14.92 ± 4.93*	0.025
Apical cavity	− 19.52 ± 4.22	− 18.30 ± 4.59	− 17.81 ± 6.68	0.227
Longitudinal Peak stain (%)				
GLS	− 16.07 ± 2.16	− 13.91 ± 2.68*	− 12.38 ± 2.69*§	< 0.001
Basal cavity	− 13.37 ± 4.54	− 12.62 ± 4.43	− 10.29 ± 4.96*§	0.02
Mid cavity	− 16.90 ± 3.42	− 14.48 ± 4.16*	− 13.31 ± 4.22*	< 0.001
Apical cavity	− 18.75 ± 2.85	− 17.10 ± 2.86*	− 15.67 ± 3.07*§	< 0.001

*GRS* global radial strain, *GCS* global circumferential strain, *GLS* global longitudinal strain

^*^p < 0.05 vs. controls

^§^p < 0.05 vs. HTN(T2DM-)

**Fig. 4:**
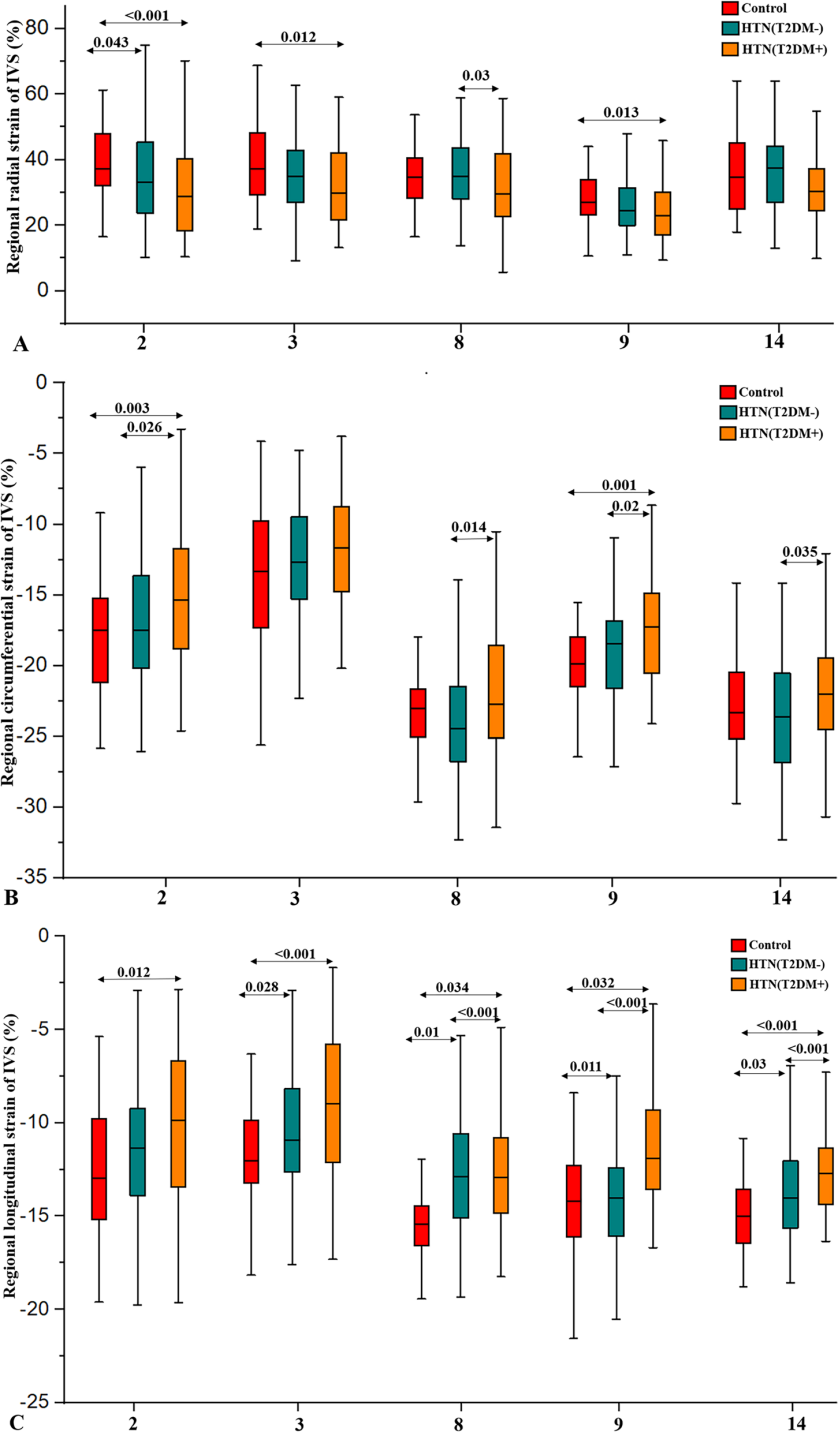
Comparisons of regional strains in segments 2, 3, 8, 9, and 14 representing the area of interventricular septum among groups. Boxplots represent the median and interquartile range for regional radial (**A**), circumferential (**B**) and longitudinal (**C**) strains of IVS segments. Significance was calculated using one-way ANOVA

As shown in Fig. 4, the regional longitudinal strains of segments 8 (− 15.57 ± 2.24 vs. − 14.04 ± 3.06 vs. − 12.79 ± 3.08%, p < 0.001), 9 (− 14.23 ± 2.79 vs. − 12.64 ± 2.98 vs. − 11.32 ± 3.22%, p < 0.001) and 14 (− 14.92 ± 2.09 vs. − 13.84 ± 2.54 vs. − 12.58 ± 2.14%, p < 0.001) decreased significantly from controls through HTN(T2DM-) to the HTN(T2DM +) groups. The regional longitudinal strains of segments 2 (− 10.36 ± 4.14 vs. − 12.56 ± 3.76%, p = 0.012) and 3 (− 9.23 ± 3.78 vs. − 12.21 ± 3.28%, p < 0.001) in the HTN(T2DM +) group and the regional longitudinal strain of segment 3 (− 10.55 ± 3.39 vs. − 12.21 ± 3.28%, p = 0.028) in the HTN(T2DM-) group were significantly reduced compared to the controls, but the value wase not significantly different between the patient groups in segment 3 (p = 0.556). The regional circumferential strains of segments 2, 8, 9 and 14 in the HTN(T2DM +) group were significantly lower than the HTN(T2DM-) group or controls (all p < 0 05).

### Correlation between ventricles in hypertension

In patients with hypertension (Table 4), the RVEF was significantly correlated with RV GCS (r = − 0.384, p < 0.001) and GRS (r = 0.294, p < 0.001) but not GLS (r = − 0.047, p = 0.585). In addition, it was correlated with all LV global strains and circumferential and longitudinal strains of the IVS. All the RV global strains correlated with LV global strains and most of the regional strains of IVS.

**Table 4:** Correlation between CMR-derived RV function and both ventricular volumetrics, LV deformation and regional strain of IVS in patients

	RVEF		RVEF		RV GCS		RV GRS	
	*r*	*p*	*r*	*p*	*r*	*p*	*r*	*p*
LV volumetrics								
LVEF	0.469	< 0.001	− 0.312	< 0.001	− 0.210	0.013	0.193	0.022
LVEDVI	− 0.285	0.001	0.050	0.566	0.084	0.328	− 0.082	0.338
VESVI	− 0.407	< 0.001	0.218	0.011	0.172	0.045	− 0.147	0.087
LVSVI	− 0.082	0.345	− 0.139	0.106	− 0.031	0.721	0.030	0.727
LV cardiac index	− 0.008	0.924	− 0.149	0.087	− 0.076	0.381	0.022	0.798
LVMI	− 0.109	0.207	0.031	0.719	0.138	0.109	− 0.036	0.674
LV remodeling index	0.117	0.166	0.026	0.756	0.146	0.084	− 0.047	0.580
RV volumetrics								
RVEF			− 0.047	0.585	− 0.384	< 0.001	0.294	< 0.001
RVEDVI	− 0.347	< 0.001	− 0.002	0.978	− 0.059	0.495	− 0.220	0.010
RVESVI	− 0.726	< 0.001	0.058	0.506	0.151	0.080	− 0.290	0.001
RVSVI	0.239	0.005	− 0.022	0.804	− 0.272	0.001	− 0.029	0.739
RV cardiac index	0.236	0.006	− 0.083	0.341	− 0.287	0.001	− 0.019	0.830
RVMI	− 0.279	0.001	0.052	0.549	0.007	0.938	− 0.104	0.229
RV remodeling index	0.146	0.083	0.061	0.473	0.113	0.183	0.195	0.021
Global strain of LV and regional strain of IVS								
LV GRS	0.469	< 0.001	− 0.306	< 0.001	− 0.322	< 0.001	0.247	0.003
LV GCS	− 0.342	< 0.001	0.292	< 0.001	0.294	< 0.001	− 0.211	0.012
LV GLS	− 0.302	< 0.001	0.393	< 0.001	0.281	0.001	− 0.208	0.013
Regional longitudinal strain of IVS								
2	− 0.338	< 0.001	0.212	0.012	0.284	0.001	− 0.318	< 0.001
3	− 0.319	< 0.001	0.314	< 0.001	0.199	0.020	− 0.237	0.005
8	− 0.274	0.001	0.313	< 0.001	0.257	0.002	− 0.179	0.034
9	− 0.275	0.001	0.340	< 0.001	0.290	< 0.001	− 0.153	0.070
14	− 0.291	0.001	0.380	< 0.001	0.232	0.006	− 0.139	0.104
Regional circumferential strain of IVS								
2	− 0.350	< 0.001	0.216	0.011	0.294	< 0.001	− 0.343	< 0.001
3	− 0.247	0.004	0.185	0.032	0.101	0.242	− 0.069	0.425
8	− 0.354	< 0.001	0.231	0.006	0.205	0.015	− 0.191	0.023
9	− 0.377	< 0.001	0.324	< 0.001	0.226	0.007	− 0.213	0.011
14	− 0.267	0.001	0.283	0.001	0.160	0.058	− 0.148	0.081

*LV* left ventricular, *RV* right ventricular, *EF* ejection fraction, *EDV* end diastolic volume, *ESV* end systolic volume, *SV* stroke volume, *M* mass, *I* indexed to BSA, *GRS* global radial strain, *GCS* global circumferential strain, *GLS* global longitudinal strain

### Associations of biventricular strains and clinical variables in the entire and patient population

After adjusting for SBP, age, sex, BMI, heart rate, and eGFR, multivariable regression analyses of the overall population showed that hypertension and T2DM were independently associated with LV GLS (β = 1.516 and 1.227, p = 0.004 and 0.009, model R^2^ = 0.374) and RV GLS (β = 2.245 and 1.328, p < 0.001 and = 0.012, model R^2^ = 0.232). T2DM, but not hypertension, was independently associated with LV GCS and GRS (β = 1.621, p = 0.004, model R^2^ = 0.305 and β = − 4.557, p = 0.003, model R^2^ = 0.263, respectively), and neither of them associated with RV GRS or GCS.

After adjusting for the above covariates, smoking and LVMI, multivariable regression analyses of patients with hypertension (Table 5) demonstrated that T2DM was independently associated with LV GRS (β = − 4.278, p = 0.004, model R^2^ = 0.285), GCS (β = 1.498, p = 0.006, model R^2^ = 0.363), GLS (β = 1.133, p = 0.007, model R^2^ = 0.372) and RV GLS (β = 1.454, p = 0.003, model R^2^ = 0.142), but not with RV GRS and GCS. When T2DM and LV GLS were included in the regression analyses, both T2DM and LV GLS (β = 0.977 and 0.362, p = 0.039 and < 0.001, model R^2^ = 0.224) were independently associated with RV GLS.

**Table 5:** Multivariate association of T2DM with both ventricular strains in all patients with hypertension adjusted for SBP, age, sex, BMI, heart rate, triglyceride, cholesterol, HDL, LDL, smoking, LVMI and eGFR

Model	GRS		GCS		GLS	
	Coefficient (95%CI)	R^2^	Coefficient (95%CI)	R^2^	Coefficient (95%CI)	R^2^
	Model 1					
T2DM	− 4.278 (− 7.191 to − 1.366) *	0.285	1.498 (0.430 to 2.566) *	0.363	1.133 (0.320 to 1.947) *	0.372
	Model 2					
T2DM	-	0.024	-	0.135	1.454 (0.511 to 2.398) *	0.142
	3					
	T2DM (−)	0.086	T2DM (−)	0.167	T2DM: 0.977 (0.051 to 1.904) *	0.224
	LV GRS: 0.302 (0.060 to 0.543) *		LV GCS: 0.340 (0.125 to 0.482) *		LV GLS: 0.362 (0.200 to 0.525) *	

Abbreviation of eGFR, HDL and LDL are shown in Table 1; LVMI: LV mass index; GRS, GCS and GLS are shown in Table 3

Model 1: Association of T2DM with LV strains

Model 2: Association of T2DM with RV strains

Model 3: Association of T2DM and LV strains with RV strains

^*^p < 0.05, values are unstandardized estimate coefficients (B) and 95% confident interval (CI)

Variables with p < 0.1 were included in the multivariable regression analyses

### Intra‑ and interobserver variability in RV strain measurement

As demonstrated in Table 6, there was excellent intraobserver (ICC: 0.860–0.954) and interobserver (ICC: 0.805–0.906) variability in the global RV measurement. Except the regional RV radial strain at the apical cavity showed good intraobserver variability (ICC = 0.726), all the other regional RV strains demonstrated excellent intraobserver variability (ICC: 0.791–0.913). The regional RV strain measurement in the basal and apical cavities showed good interobserver variability (ICC: 0.643–0.716), and the regional strain measurement in the middle cavity demonstrated excellent interobserver variability (ICC: 0.754–0.805).

**Table 6:** Intra-and inter-observer variability of global RV and regional strains

	Intra-observer		Inter-observer	
	ICC	95%CI	ICC	95%CI
Radial peak strain				
Global	0.86	0.726–0.931	0.805	0.629–0.902
Basal cavity	0.815	0.648–0.908	0.704	0.465–0.847
Mid cavity	0.878	0.760–0.940	0.755	0.546–0.875
Apical cavity	0.726	0.500–0.860	0.688	0.44–0.838
Circumferential peak strain				
Global	0.946	0.890–0.974	0.904	0.808–0.953
Basal cavity	0.867	0.740–0.930	0.716	0.484–0.854
Mid cavity	0.913	0.826–0.958	0.805	0.647–0.907
Apical cavity	0.943	0.883–0.972	0.643	0.373–0.813
Longitudinal peak strain				
Global	0.954	0.906–0.978	0.906	0.812–0.954
Basal cavity	0.876	0.757–0.939	0.715	0.483–0.854
Mid cavity	0.91	0.820–0.956	0.754	0.545–0.875
Apical cavity	0.791	0.606–0.895	0.69	0.443–0.839

*ICC* intraclass correlation coefficient,* CI* confidence interval.

## Discussion

The present study used the relatively new technique CMR-FT to evaluate the effect of T2DM on global and regional RV myocardial strains in patients with essential hypertension and explore the relationship between RV function and that of left ventricle and IVS. Our results demonstrated that the biventricular GLS and regional longitudinal strain of the right ventricle and IVS decreased significantly in patients with hypertension and was further deteriorated by T2DM. The RV global strains correlated with that of left ventricle and regional strain of IVS in patients. LV GLS impairment superimposed by coexisting T2DM was independently associated with RV GLS in patients with hypertension, which suggests an adverse interaction between ventricles.

### RV systolic dysfunction in hypertension

Previous CMR studies have demonstrated RV hypertrophy and remodeling characterized by an increased RVMI and remodeling index in patients with hypertension [21, 22], which is consistent with our results. In addition, some previous echocardiographic studies have revealed increased RV wall thickness and remodeling in hypertension [14–16]. There are obvious limitations in utilizing the ejection fraction to evaluate cardiac systolic function in cases of ventricular hypertrophy [23] because ventricular load affects its measurement [8]. Kareye et al. found that approximately 33% of patients with hypertensive heart disease had impairment of RV systolic function, which was defined as tricuspid annulus plane systolic displacement less than 15 mm [24]. The myocardial strain and strain rate may be used to directly evaluate myocardial function because these measurements are not theoretically affected by the size or shape of the cardiac chamber. The subendocardial fibers of the right ventricle are arranged longitudinally, but the subepicardial fibers circumferentially. During RV contraction, longitudinal shortening accompanied by the movement of myocardial fibers toward the apex of the heart is more significant than circumferential shortening [25], and it is the main determinant of RVEF [26]. In our patients with hypertension and preserved RVEF, the RVEF was associated with RV GCS but not RV GLS, which may suggest that the RV GCS plays an important role in maintaining normal RVEF when RV GLS was reduced.

A previous study has showed that the longitudinal strain was an independent predictor for RV systolic dysfunction [27], which was associated with morbidity and mortality in a variety of cardiovascular diseases [28, 29]. Impairment of RV longitudinal strain may occur in these diseases and show a progressive decline in the early stage, but the circumferential strain, which represents the function of circumferential fibers in the subepicardial layer, did not decrease or even increased [26], which is consistent with the decrease of RV longitudinal strain in our patients Using two-dimensional echocardiography strain analysis, previous studies showed a decrease in RV peak systolic strain in patients with treated [13]and untreated hypertensive patients [14, 15]. In addition, there were reduced RV global longitudinal strain and systolic strain rate in untreated and uncontrolled hypertensive patients compared with the controls and well-controlled patients [16], even in patients with high-normal blood pressure [17].Therefore, we speculate that the RV systolic dysfunction was presented in hypertensive patients with preserved RVEF, and the longitudinal strain is a sensitive indicator of RV systolic dysfunction in the early stage.

### T2DM aggravates RV systolic dysfunction in hypertension

Cardiovascular complications are important causes of diabetes-associated morbidity and mortality. T2DM leads to myocardial dysfunction that often exhibits no obvious symptoms in the early stage but progresses to obvious diabetic cardiomyopathy in the absence of timely and adequate treatment. RV dysfunction is an important component of diabetic cardiomyopathy, and several previous studies have showed decreased RV longitudinal strain in patients with T2DM [30–32]. Our study found that the global RV strain and regional strains of right ventricle and IVS were decreased in patients with HTN(T2DM +) compared to patients with HTN(T2DM−), which suggests that coexisting T2DM further exaggerates the RV systolic dysfunction in hypertension.

Hearts in patients with T2DM are susceptible to atherosclerosis, subclinical micromyocardial infarction, advanced glycosylation end-product (AGE) deposition, mitochondrial dysfunction and lipid toxicity [33]. Excessive triglycerides in cardiomyocytes lead to myocardial steatosis, which impairs the systolic function of the RV myocardium [31]. A recent animal experiment showed that reducing myocardial fat accumulation improved myocardial cell function [34]. Our previous study showed that coexisting T2DM exacerbated LV systolic dysfunction in patients with hypertension via superimposed impairment of LV myocardial microcirculation [35]. Evaluating the myocardial perfusion of right ventricle is difficult due to its thin myocardial wall, and it was not performed in our study. We postulated that the microcirculation of RV myocardium was impaired in our patients, which needs to be validated in further study. Linssen et al. [6] found that the RV systolic and diastolic function in patients with diabetes were not associated with those of left ventricle, which suggests that diabetes directly impairs RV function. However, we found that T2DM was associated with the decline of RV GLS by superposing impairment to the LV GLS in patients with hypertension. CMR-FT directly evaluates the function of myocardium at the myocardial level, then we hypothesized that T2DM can not only directly impair the RV systolic function but also lead to RV dysfunction by impairing the function of left ventricle and IVS.

### Interaction between ventricles

Animal experiments showed that approximately 20–40% of RV output was related to the contractile effect of left ventricle [36]. The right ventricle is not directly exposed to systemic pressure, even without an increase in RV load due to increased LV diastolic pressure [8], the mechanism of RV dysfunction in patients with hypertension is not clear. The present study found that the RV global strains were closely correlated with those of left ventricle and regional strains of IVS in patients with hypertension, and the decreased RV GLS was associated with the superimposed impairment of LV GLS by the coexisting T2DM. Our study confirmed previous echocardiographic results that the RV systolic function defined by tricuspid annulus systolic displacement was associated with LV long-axis function and mitral annular plane lateral and septal wall systolic displacement [24]. These results suggest that RV disease progression was consistent with that of left ventricle in patients with hypertension, which may be due to the adverse interventricular interactions in which IVS played an important role.

Interventricular interaction was defined as the transfer of force from one ventricle to the other through the myocardium and pericardium, which is unrelated to the neurological, humoral and circulatory effects [37]. Some studies speculated that the interaction between ventricles was due to their close anatomical relationship, i.e., they are surrounded by common myocardial fibers, have a common IVS and show limited interventricular septal displacement in the pericardial cavity [23, 37]. Notably, the IVS may play a vital role because it is involved in the ejection and filling of the right ventricle [38].

## Limitations

There are some shortcomings in this study. First, this was a cross-sectional single-center study with a relatively small sample size, and selective bias may be existed. Further longitudinal multicentric large sample studies are needed to confirm our results. Second, the effect of hypertension on pulmonary circulation was not evaluated in our study, whether there was an increase in RV afterload and its effect on RV function could not be determined which needs further investigation. Third, animal experiments were not performed in our study, and relevant pathological mechanisms will be investigated in future studies. Finally, follow-up was not performed to evaluate the prognostic value of RV dysfunction, but these studies would provide important information for the prevention and improvement of RV dysfunction.

## Conclusions

T2DM may exacerbate RV systolic dysfunction in patients with hypertension, which may be associated with the superimposed global LV and regional IVS dysfunction by the coexisting T2DM. These results suggest an adverse interventricular interaction.

## Data Availability

The datasets used and analyzed during the current study are available from the corresponding author on reasonable request.

## Abbreviations

HTN: Hypertension
T2DM: Type to diabetes mellitus
LV: Left ventricular
RV: Right ventricular
CMR: Cardiovascular magnetic resonance
CMR-FT: CMR feature tracking
EDV: End-diastolic volume
ESV: End-diastolic volume
SV: Stroke volume
CO: Cardiac output
EF: Ejection fraction
LVMI: LV mass index
GRS: Global radial strain
GCS: Global circumferential strain
GLS: Global longitudinal strain
IVS: Interventricular septum
